# Diethyl 2,2′-bis­(hy­droxy­imino)-3,3′-(hydrazinediyl­idene)dibutano­ate

**DOI:** 10.1107/S1600536812007398

**Published:** 2012-02-24

**Authors:** Hui Li, Peng-Fei Su, Jun-Feng Tong, Bo-Zhou Wang

**Affiliations:** a Xi’an Modern Chemistry Research Institute, Xi’an 710065, People’s Republic of China; b Key Labortary of Opto-Electronic Technology and Intelligent Control (Lanzhou Jiaotong University), Ministry of Education, Lanzhou 730070, People’s Republic of China

## Abstract

Each mol­ecule of the title compound, C_12_H_18_N_4_O_6_, is located on an inversion centre at the mid-point of the central N—N bond. The azo groups C=N of the Schiff base group have an *E* conformation and the azo groups in the oxime C=N—O groups have a *Z* conformation. O–H⋯O hydrogen bonds link neighbouring mol­ecules into infinite monolayers perpendicular to the *a* axis.

## Related literature

For background to strobilurin A and strobilurin analogs, see: Zhao *et al.* (2007 ▶); Balbaa (2007 ▶); Li *et al.* (2010 ▶); Zakharychev *et al.* (1999 ▶, 2001 ▶). For bond-length data, see: Allen *et al.* (1987 ▶).
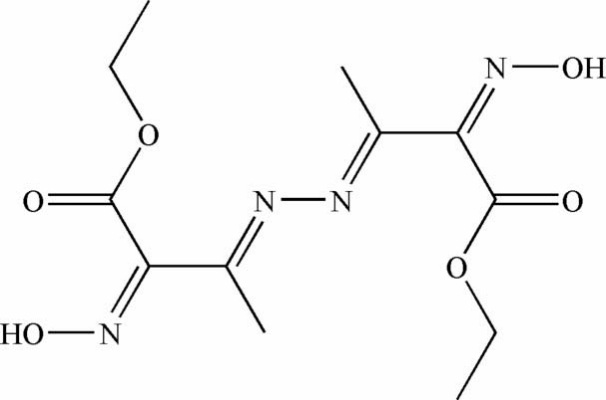


## Experimental

### 

#### Crystal data

C_12_H_18_N_4_O_6_
*M* *_r_* = 314.30
Monoclinic, 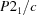

*a* = 10.8587 (11) Å
*b* = 8.3068 (9) Å
*c* = 8.8465 (9) Å
β = 99.782 (2)°
*V* = 786.36 (14) Å^3^
*Z* = 2
Mo *K*α radiation
μ = 0.11 mm^−1^
*T* = 296 K
0.18 × 0.18 × 0.10 mm

#### Data collection

Bruker APEXII CCD diffractometer
3779 measured reflections
1385 independent reflections
1235 reflections with *I* > 2σ(*I*)
*R*_int_ = 0.015

#### Refinement

*R*[*F* ^2^ > 2σ(*F* ^2^)] = 0.039
*wR*(*F* ^2^) = 0.110
*S* = 1.09
1385 reflections
103 parameters
H-atom parameters constrained
Δρ_max_ = 0.20 e Å^−3^
Δρ_min_ = −0.24 e Å^−3^

### 

Data collection: *APEX2* (Bruker, 2007 ▶); cell refinement: *SAINT* (Bruker, 2007 ▶); data reduction: *SAINT*; program(s) used to solve structure: *SHELXS97* (Sheldrick, 2008 ▶); program(s) used to refine structure: *SHELXL97* (Sheldrick, 2008 ▶); molecular graphics: *SHELXTL* (Sheldrick, 2008 ▶); software used to prepare material for publication: *SHELXTL*.

## Supplementary Material

Crystal structure: contains datablock(s) global, I. DOI: 10.1107/S1600536812007398/zl2446sup1.cif

Structure factors: contains datablock(s) I. DOI: 10.1107/S1600536812007398/zl2446Isup2.hkl

Supplementary material file. DOI: 10.1107/S1600536812007398/zl2446Isup3.cml

Additional supplementary materials:  crystallographic information; 3D view; checkCIF report

## Figures and Tables

**Table 1:** Hydrogen-bond geometry (Å, °)

*D*—H⋯*A*	*D*—H	H⋯*A*	*D*⋯*A*	*D*—H⋯*A*
O3—H3⋯O2^i^	0.82	1.95	2.7577 (15)	170

Symmetry code: (i) 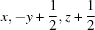.

**Fig. 1.**
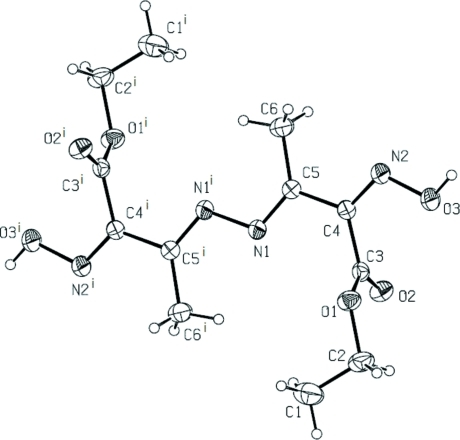
The molecular structure of the title compound with the atom numbering scheme [symmetry code: i) -x, -y, -z]. Displacement ellipsoids for non-hydrogen atoms are drawn at the 30% probability level.

**Fig. 2.**
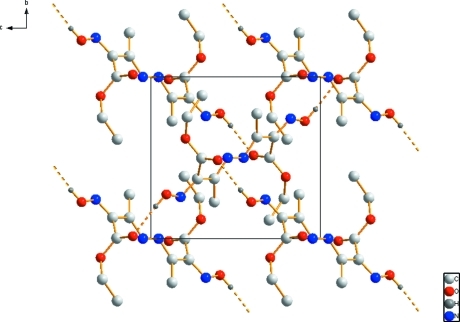
Intermolecular hydrogen bonding of the title compound viewed along the a axis. Hydrogen atoms not involved in hydrogen bonding have been omitted for clarity.

**Fig. 3.**
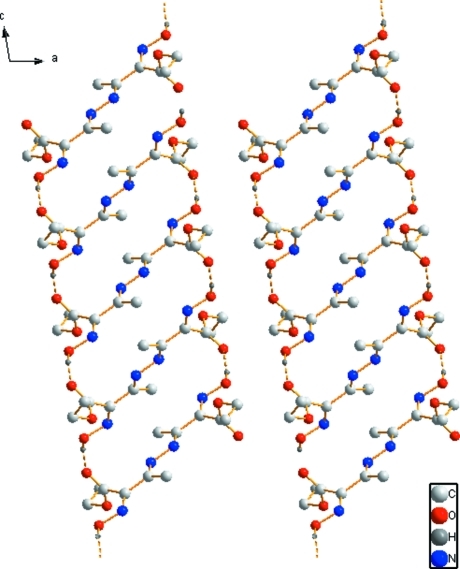
View of intermolecular hydrogen bonding, showing sections of two infinite two-dimensional monolayers viewed along the b axis. H atoms not involved in hydrogen bonding have been omitted for clarity.

## supplementary crystallographic information

### Comment

The strobilurins (Balbaa, 2007; Li *et al.*, 2010; Zhao *et al.*, 2007) are one of the most important classes of agricultural fungicides, with Strobilurin A as the lead compound, which inhibit electron transfer in mitochondria, disrupt metabolism and prevent growth of the target fungi. Reaction of 2-hydroxyimino-3-oxobutanoic acid esters with hydrazine hydrate can afford a product with a structure close to that of Strobilurin A (Zakharychev *et al.*, 1999; Zakharychev *et al.*, 2001). Herein, we report the synthesis and crystal structure of this compound, diethyl-2,2'-(hydroxyimino)-3,3'-azino-di-butanoate.

A perspective view of the title compound, showing the atomic numbering scheme, is given in Fig. 1. The complete molecule of the title compound is located on an inversion centre at the mid-point of the the central N—N bond. The azo groups in Schiff base C═N group are in *E* configuration and the azo groups in the oxime C═N—O groups are in *Z* configuration. The bond lengths (Allen *et al.*, 1987) and angles in the molecule are within normal ranges. In each molecule, twelve atoms including C4, C4^i^, C5, C5^i^, C6, C6^i^, N1, N1^i^, N2, N2^i^, O3, O3^i^ (symmetry code: i = -*x*, -*y*, -*z*) are coplanar with little deviation from their mean plane of 0.090 (3), 0.039 (4), 0.026 (3), 0.030 (1), 0.088 (5), and 0.007 (1) Å, respectively, and the deviation from the mean plane of other atoms C1, C1^i^, C2, C2^i^, C3, C3^i^, O1, O1^i^, and O2, O2^i^ are 0.778 (2), 0.545 (4), 0.396 (5), 0.691 (1) and 1.523 (7) Å, respectively. Meanwhile, the dihedral angle between the plane defined by C2, C3, C4, O1 and O2 and the main plane is 87.87 (2) °.

In the crystal environment of each molecule of the title compound, there exist four symmetry equivalent intermolecular O3—H3···O2 hydrogen bonds (Table 1, Fig. 2), two of which originate from the each molecule and two for which the molecule acts as the H bonding acceptor unit. Each molecule links neighbouring molecules into an infinite monolayer perpendicular to the *a* axis (Fig. 3).

### Experimental

To an acetic acid (32 ml) solution of ethyl acetoacetate (15 g) was added a solution of sodium nitrite in water (25 ml) at 263 K, and the reaction mixture was poured to ice-water (100 ml), then the mixture was extracted three times with ether (50 ml). The extracts were washed with water, after which the solvent was distilled off to give a light yellow residue. This residue was dissolved in methanol (20 ml) and the solution was added dropwise to a mixture of hydrazine hydrate (2.9 g, 85%), methanol (50 ml) and water (37 ml) at 273 K, and the resulting mixture was stirred for six hours at the same temperature. The reaction mixture was extracted two times with ethyl acetate (50 ml), then the extracts were washed with water and dried over magnesium sulfate, filtered and the solvent was removed to give the crude product. The title compound was purified by column chromatography on silica gel using a mixture of dichloromethane and methanol (*R*_f_ = 0.35, 20:1, V/V) as the eluent, affording the title compound 4.71 g. Yield, 26.1%. m. p. 473–475 K. ^1^H NMR (d_6_-DMSO): 1.24 (t, 6H, ^3^J = 7.10 Hz), 1.96 (s, 6H), 4.25 (q, 4H, ^3^J = 7.08 Hz), 12.54 (s, 2H).

Pale yellow block-like single crystals of the title compound suitable for X-ray diffraction studies were obtained after three weeks by slow evaporation from a mixture of dichloromethane and methanol at room temperature.

### Refinement

The carbon-bound H-atoms were positioned geometrically and included in the refinement using a riding model with distances C—H = 0.96 Å (CH_3_) and 0.97 Å (CH_2_). And the oxygen-bound H-atoms were located in difference Fourier maps and refined with an O—H distance constrained to 0.85 Å. The isotropic displacement parameters for all H atoms were set equal to 1.2 *U*_eq_ (for CH_2_) or 1.5 *U*_eq_ (for CH_3_ and OH) of the carrier atom.

### Crystal data

**Table:**

C_12_H_18_N_4_O_6_	*F*(000) = 332
*M**_r_* = 314.30	*D*_x_ = 1.327 Mg m^−^^3^
Monoclinic, *P*2_1_/*c*	Melting point = 473–475 K
Hall symbol: -P 2ybc	Mo *K*α radiation, λ = 0.71073 Å
*a* = 10.8587 (11) Å	Cell parameters from 2318 reflections
*b* = 8.3068 (9) Å	θ = 3.1–28.2°
*c* = 8.8465 (9) Å	µ = 0.11 mm^−^^1^
β = 99.782 (2)°	*T* = 296 K
*V* = 786.36 (14) Å^3^	Block-like, yellow
*Z* = 2	0.18 × 0.18 × 0.10 mm

### Data collection

**Table:**

Bruker APEXII CCD diffractometer	1235 reflections with *I* > 2σ(*I*)
Radiation source: fine-focus sealed tube	*R*_int_ = 0.015
Graphite monochromator	θ_max_ = 25.0°, θ_min_ = 1.9°
φ and ω scans	*h* = −6→12
3779 measured reflections	*k* = −9→9
1385 independent reflections	*l* = −10→10

### Refinement

**Table:**

Refinement on *F*^2^	Primary atom site location: structure-invariant direct methods
Least-squares matrix: full	Secondary atom site location: difference Fourier map
*R*[*F*^2^ > 2σ(*F*^2^)] = 0.039	Hydrogen site location: inferred from neighbouring sites
*wR*(*F*^2^) = 0.110	H-atom parameters constrained
*S* = 1.09	*w* = 1/[σ^2^(*F*_o_^2^) + (0.0587*P*)^2^ + 0.202*P*] where *P* = (*F*_o_^2^ + 2*F*_c_^2^)/3
1385 reflections	(Δ/σ)_max_ = 0.001
103 parameters	Δρ_max_ = 0.20 e Å^−^^3^
0 restraints	Δρ_min_ = −0.24 e Å^−^^3^

### Special details

**Table:**

Geometry. All e.s.d.'s (except the e.s.d. in the dihedral angle between two l.s. planes) are estimated using the full covariance matrix. The cell e.s.d.'s are taken into account individually in the estimation of e.s.d.'s in distances, angles and torsion angles; correlations between e.s.d.'s in cell parameters are only used when they are defined by crystal symmetry. An approximate (isotropic) treatment of cell e.s.d.'s is used for estimating e.s.d.'s involving l.s. planes.
Refinement. Refinement of *F*^2^ against ALL reflections. The weighted *R*-factor *wR* and goodness of fit *S* are based on *F*^2^, conventional *R*-factors *R* are based on *F*, with *F* set to zero for negative *F*^2^. The threshold expression of *F*^2^ > σ(*F*^2^) is used only for calculating *R*-factors(gt) *etc*. and is not relevant to the choice of reflections for refinement. *R*-factors based on *F*^2^ are statistically about twice as large as those based on *F*, and *R*- factors based on ALL data will be even larger.

### Fractional atomic coordinates and isotropic or equivalent isotropic displacement parameters (Å^2^)

**Table:**

	*x*	*y*	*z*	*U*_iso_*/*U*_eq_	
C1	0.3173 (2)	−0.3855 (3)	0.1974 (3)	0.0815 (7)	
H1A	0.2823	−0.3486	0.0964	0.122*	
H1B	0.2514	−0.4229	0.2485	0.122*	
H1C	0.3745	−0.4720	0.1899	0.122*	
C2	0.38401 (17)	−0.2522 (2)	0.2854 (2)	0.0543 (5)	
H2A	0.4193	−0.2890	0.3877	0.065*	
H2B	0.4520	−0.2159	0.2355	0.065*	
C3	0.29765 (13)	0.00236 (17)	0.20059 (16)	0.0335 (3)	
C4	0.20477 (13)	0.12983 (16)	0.22471 (16)	0.0350 (4)	
C5	0.08145 (13)	0.12936 (17)	0.12596 (17)	0.0364 (4)	
C6	−0.00087 (18)	0.2722 (2)	0.1254 (3)	0.0702 (6)	
H6A	−0.0799	0.2511	0.0615	0.105*	
H6B	0.0377	0.3636	0.0862	0.105*	
H6C	−0.0137	0.2943	0.2281	0.105*	
O1	0.29746 (10)	−0.11865 (13)	0.29545 (13)	0.0455 (3)	
O2	0.36466 (10)	0.01499 (13)	0.10565 (12)	0.0442 (3)	
O3	0.34902 (10)	0.22632 (15)	0.40794 (13)	0.0518 (4)	
H3	0.3626	0.2998	0.4704	0.078*	
N2	0.22952 (12)	0.24201 (15)	0.32449 (15)	0.0431 (4)	
N1	0.05904 (11)	0.00033 (14)	0.04649 (14)	0.0372 (3)	

### Atomic displacement parameters (Å^2^)

**Table:**

	*U*^11^	*U*^22^	*U*^33^	*U*^12^	*U*^13^	*U*^23^
C1	0.0890 (16)	0.0520 (12)	0.0992 (17)	0.0159 (11)	0.0036 (13)	−0.0098 (11)
C2	0.0521 (10)	0.0444 (10)	0.0662 (11)	0.0153 (7)	0.0097 (9)	0.0135 (8)
C3	0.0317 (7)	0.0335 (8)	0.0333 (7)	−0.0029 (6)	−0.0005 (6)	−0.0018 (5)
C4	0.0343 (8)	0.0335 (8)	0.0370 (7)	−0.0025 (6)	0.0056 (6)	−0.0020 (6)
C5	0.0333 (8)	0.0356 (8)	0.0399 (8)	0.0008 (6)	0.0051 (6)	−0.0025 (6)
C6	0.0517 (11)	0.0600 (12)	0.0899 (15)	0.0196 (9)	−0.0140 (10)	−0.0303 (11)
O1	0.0461 (7)	0.0393 (6)	0.0530 (7)	0.0064 (5)	0.0133 (5)	0.0100 (5)
O2	0.0430 (6)	0.0473 (7)	0.0441 (6)	0.0072 (5)	0.0123 (5)	0.0068 (5)
O3	0.0424 (6)	0.0549 (7)	0.0529 (7)	−0.0011 (5)	−0.0065 (5)	−0.0187 (5)
N2	0.0381 (7)	0.0438 (8)	0.0458 (7)	−0.0014 (5)	0.0026 (6)	−0.0101 (6)
N1	0.0318 (6)	0.0352 (7)	0.0420 (7)	−0.0003 (5)	−0.0012 (5)	−0.0008 (5)

### Geometric parameters (Å, º)

**Table:**

C1—C2	1.472 (3)	C4—N2	1.2803 (19)
C1—H1A	0.9600	C4—C5	1.469 (2)
C1—H1B	0.9600	C5—N1	1.2822 (19)
C1—H1C	0.9600	C5—C6	1.485 (2)
C2—O1	1.4663 (19)	C6—H6A	0.9600
C2—H2A	0.9700	C6—H6B	0.9600
C2—H2B	0.9700	C6—H6C	0.9600
C3—O2	1.2056 (17)	O3—N2	1.3857 (17)
C3—O1	1.3097 (18)	O3—H3	0.8200
C3—C4	1.5024 (19)	N1—N1^i^	1.401 (2)
			
C2—C1—H1A	109.5	N2—C4—C3	122.92 (13)
C2—C1—H1B	109.5	C5—C4—C3	118.66 (12)
H1A—C1—H1B	109.5	N1—C5—C4	113.40 (12)
C2—C1—H1C	109.5	N1—C5—C6	127.44 (14)
H1A—C1—H1C	109.5	C4—C5—C6	119.15 (13)
H1B—C1—H1C	109.5	C5—C6—H6A	109.5
O1—C2—C1	109.77 (16)	C5—C6—H6B	109.5
O1—C2—H2A	109.7	H6A—C6—H6B	109.5
C1—C2—H2A	109.7	C5—C6—H6C	109.5
O1—C2—H2B	109.7	H6A—C6—H6C	109.5
C1—C2—H2B	109.7	H6B—C6—H6C	109.5
H2A—C2—H2B	108.2	C3—O1—C2	118.11 (12)
O2—C3—O1	125.45 (13)	N2—O3—H3	109.5
O2—C3—C4	122.47 (13)	C4—N2—O3	111.52 (12)
O1—C3—C4	112.07 (12)	C5—N1—N1^i^	113.25 (14)
N2—C4—C5	118.38 (13)		
			
O2—C3—C4—N2	−93.59 (18)	O2—C3—O1—C2	−1.0 (2)
O1—C3—C4—N2	85.26 (17)	C4—C3—O1—C2	−179.80 (13)
O2—C3—C4—C5	84.22 (17)	C1—C2—O1—C3	−99.25 (19)
O1—C3—C4—C5	−96.93 (15)	C5—C4—N2—O3	−179.79 (12)
N2—C4—C5—N1	−170.22 (14)	C3—C4—N2—O3	−2.0 (2)
C3—C4—C5—N1	11.88 (19)	C4—C5—N1—N1^i^	179.46 (13)
N2—C4—C5—C6	10.7 (2)	C6—C5—N1—N1^i^	−1.6 (3)
C3—C4—C5—C6	−167.18 (16)		

Symmetry code: (i) −*x*, −*y*, −*z*.

### Hydrogen-bond geometry (Å, º)

**Table:**

*D*—H···*A*	*D*—H	H···*A*	*D*···*A*	*D*—H···*A*
O3—H3···O2^ii^	0.82	1.95	2.7577 (15)	170

Symmetry code: (ii) *x*, −*y*+1/2, *z*+1/2.
